# Glutamate Supplementation Improves Growth Performance, Rumen Fermentation, and Serum Metabolites in Heat-Stressed Hu Sheep

**DOI:** 10.3389/fnut.2022.851386

**Published:** 2022-04-06

**Authors:** Chuan Li, Jiantong Zhang, Yanjiao Li, Xianghui Zhao, Huan Liang, Kairong Li, Mingren Qu, Qinghua Qiu, Kehui Ouyang

**Affiliations:** ^1^Jiangxi Province Key Laboratory of Animal Nutrition, Animal Nutrition and Feed Safety Innovation Team, College of Animal Science and Technology, Jiangxi Agricultural University, Nanchang, China; ^2^Ganzhou Lvlinwan Agriculture and Animal Husbandry Co. Ltd., Ganzhou, China

**Keywords:** glutamate, growth performance, immunity, rumen fermentation, serum metabolites, heat stress, Hu sheep

## Abstract

This study evaluated the effect of glutamate supplementation on the physiological parameters of heat-stressed Hu sheep. Forty-eight male Hu sheep with an average initial body weight of 17.74 ± 0.17 kg were randomly divided into two groups: The control group (CON) was fed a basal diet and a treatment group (GLU) was fed a basal diet + 3 g/head/day of L-glutamate. There were six replications in each group with four sheep in each replication for a 90 days feeding test. Growth performance, serum biochemistry, and serum hormones were measured during phase 1 (1–30 days), phase 2 (31–60 days), and phase 3 (61–90 days) of the experiment; rumen fermentation characteristics, nutrient digestibility, and slaughter performance were measured at the end of the experimental periods. There were no differences in growth performance, serum biochemical indices, and immune indices between CON and GLU during phases 1 and 2. However, a higher average daily gain (ADG), a lower average daily feed intake (ADFI), and a lower F:G ratio (ADFI/ADG) were observed in GLU during phase 3 (*p* < 0.05). Serum levels of glutamate, globulin, immunoglobulin A, immunoglobulin G, immunoglobulin M, and growth hormone in GLU were higher than those in CON only on day 90 (*p* < 0.05). Serum levels of heat shock protein 70, adrenocorticotrophic hormone, corticosterone, triiodothyronine, and tetraiodothyronine in GLU were lower than those in CON on day 90 (*p* < 0.05). At the end of the experiment, ruminal pH, microbial crude protein, ammonia nitrogen, and isovalerate concentrations in GLU were higher than those in CON (*p <* 0.05). The apparent digestibility of dry matter, organic matter, and crude protein in GLU was higher than those in CON (*p <* 0.05). There were no differences in carcass traits and organ indices but spleen weight and spleen index tended to be higher in GLU. In conclusion, dietary glutamate supplementation improved rumen fermentation, increased nutrition digestibility and metabolism, enhanced immunity, and promoted growth performance of heat-stressed Hu sheep. This suggests that a longer period of glutamate supplementation (not less than 60 days) at a level of 3 g/head/day is beneficial to Hu sheep under heat stress.

## Introduction

Hu sheep, commonly farmed in southern China, are renowned for their high fecundity and early sexual maturity (1). Heat stress decreases livestock productivity and fertility, and increases the disease susceptibility and mortality (2), making it one of the most difficult challenges in Hu sheep management in southern China. Heat stress inhibits protein synthesis and increases protein hydrolysis in animals (3). Degraded muscle protein supplies energy by providing amino acid (AA) substrates delivered to the liver in a process called gluconeogenesis (4). Heat stress decreases the plasma AA content (5) and increases the loss of endogenous intestinal proteins and AAs (6). Therefore, AA supplementation may be a beneficial approach to relieve heat stress in animals.

Glutamate is a functional AA. As the central hub of AA nitrogen exchange, it promotes the synthesis and degradation of proteins (7) and participates in cellular signal transduction, gene expression regulation, and metabolic cascades (8, 9). Glutamate enhances the intestinal and immune function of suckling and weaned piglets (10), and improves the productivity of pubertal rams and nulliparous ewes (11). However, the function of glutamate may be related to the physiological conditions and dosage of the supplement (10). A previous study has shown that glutamate is beneficial for enhancing the thermodynamic stability of protein ribonuclease A and α-lactalbumin for resisting temperature stress (12). Glutamate requirements are increased in heat-stressed broilers, therefore, supplementation of glutamate or its decarboxylated products (γ-GABA) can improve the intestinal morphology and survivability of broilers under heat stress (13, 14). Generally, AAs can be synthesized by rumen microorganisms and the supplementation of AAs to ruminants is unnecessary. However, under heat stress, the absorption and utilization of AAs are altered in ruminants, resulting in their reduced availability (15). In ruminants, glutamate may help to stimulate the rumen microbial growth *in vitro*, whereas deficiency of glutamate reduces the rumen microbial growth and efficiency (16, 17). Further, glutamate plays a vital role in generating α-ketoglutarate in the tricarboxylic acid cycle of rumen epithelial and duodenal mucosal cells in beef cattle (18). Glutamate may relieve heat-stress-induced intestinal epithelial cell damage in dairy cattle (19). However, little research has focused on the application effect of glutamate supplementation on ruminants, especially on sheep under heat stress.

We hypothesized that glutamate mitigates heat stress and improves the growth performance in Hu sheep under heat stress. The anti-heat stress effects of dietary glutamate supplementation on growth performance, nutrient digestibility, rumen fermentation characteristics, serum metabolites, and slaughter performance of heat-stressed Hu sheep were investigated, aiming to evaluate the application effect and mechanism of glutamate in the production of Hu sheep under heat stress.

## Materials and Methods

### Animals and Experimental Design

A total of forty-eight male Hu sheep with an average initial body weight of 17.74 ± 0.17 kg were randomly separated into a control group and a treatment group. The control group (CON) was fed a basal diet and the treatment group (GLU) was fed a basal diet + 3 g/head/d of L-glutamate. The L-glutamate was purchased from Shanghai Yuanye Biological Co. (Shanghai, China) and had a purity of 99%. The supplementation dosage of L-glutamate was estimated according to an *in vitro* pilot experiment (unpublished). The basal diet (Table 1) consisted of a complete mixed diet with a concentrate to forage ratio of 7:3 designed to meet the Chinese Feeding Standard of Sheep (NY/T816-2004) requirements for growing-sheep. Each treatment group contained twenty-four sheep divided into six replicates of four sheep.

**TABLE 1:** Composition and nutrient levels of experiment basal diets (air-dry basis, %)^a^.

Ingredients		Chemical composition	
Peanut straw, %	30.00	DM, %	93.74
Corn, %	49.38	OM, %	86.05
Wheat bran, %	1.82	ME^c^, (MJ/kg)	10.84
Soybean meal, %	14.40	CP, %	14.66
CaHPO_4_, %	0.15	NDF, %	25.97
NaHCO_3_, %	0.25	ADF, %	14.69
NaCl, %	0.50	SCHO^d^, %	41.35
Premix^b^, %	3.50	Ash, %	7.69
Total, %	100.00	Ca, %	0.50
		P, %	0.29

*^a^DM, dry matter; OM, organic matter; ME, metabolizable energy; CP, crude protein; EE, ether extract; NDF, neutral detergent fiber; ADF, acid detergent fiber; SCHO, soluble carbohydrates; Ca, calcium; P, phosphorus.*

*^b^Premix provided for 1 kg of complete diet: Cu as copper sulfate, 250 mg; Fe as iron sulfate, 1,400 mg; Zn as zinc oxide, 1,200 mg; Mn as manganese oxide, 900 mg; vitamin D3, 27 000 IU; vitamin A, 100,000 IU; vitamin E, 800 IU.*

*^c^ME was a calculated value.*

*^d^Calculated from analyzed nutrient values as 100 − (CP + EE + Ash + NDF).*

The trial lasted for 90 days from 1 June to 29 August 2021. Animals were fed twice a day (8:00 and 17:00 h) with free access to water and feed. The feed intake of each replicate was recorded daily, and the body weight of sheep was recorded after fasting for 12 h on days 0, 30, 60, and 90. The feed efficiency was calculated using the F:G ratio based on the average daily feed intake (ADFI) and average daily gain (ADG).

### Determination of Temperature and Humidity Index

Temperature and humidity were recorded by an Apresys^®^ instrument (179-TH, Apresys International Inc., San Ramon, CA, United States), and the temperature and humidity index (*THI*) was calculated according to NRC (1971) (20) as follows:


T⁢H⁢I=(1.8×A⁢T+32)-[(0.55-0.0055×R⁢H)×(1.8×T-26.8)],

where *AT* is air temperature (^°^C) and *RH* is relative humidity (%).

### Serum Sampling and Analysis

Blood samples were collected with non-anticoagulation vacuum blood vessels before morning feeding after fasting for 12 h on days 30, 60, and 90. Serum was harvested following centrifugation at 3,000 × *g* for 10 min at 4^°^C and subsequently frozen at −80^°^C until analysis.

The concentrations of serum glucose, blood urea nitrogen (BUN), total protein (TP), albumin (ALB), glutamate, and globulin (GLB) were determined using corresponding commercial kits (Zhongsheng Beikong Bio-technology and Science Inc., Beijing, China) and an automatic biochemical analyzer (BS-420, Shenzhen Mindray Bio-medical Electronics Co., Shenzhen, China). The concentrations of glutamate, immunoglobulin A (IgA), immunoglobulin G (IgG), and immunoglobulin M (IgM) were measured using corresponding commercial kits (Beijing Sino-UK Institute of Biological Technology, Beijing, China) and a semi-automatic biochemical analyzer (A6, Beijing Songshang Technology Co., Beijing, China). The concentrations of growth hormone (GH), heat shock protein 70 (HSP70), adrenocorticotrophic hormone (ATCH), corticosterone (CORT), triiodothyronine (T_3_), and tetraiodothyronine (T_4_) were determined using corresponding commercial ELISA kits (Beijing Sino-UK Institute of Biological Technology, Beijing, China) and a microplate reader (DR-200BS, Wuxi Hua Wei De Lang Instrument Co., Wuxi, China).

### Determination of Rumen Fermentation Characteristics

Rumen fluid pH was measured immediately after slaughter using a portable pH meter [Testo 206-pH1, Testo Instruments International (shanghai) CO., Shanghai, China]. Then samples were collected for the determination of microbial crude protein (MCP), ammonia nitrogen (NH_3_-N), and volatile fatty acids (VFAs). The VFA concentration was analyzed by gas chromatography (GC-2014, Shimadzu, Tokyo, Japan) equipped with a capillary column (Stabilwax, Restek, Bellefonte, PA, United States). NH_3_-N concentration was determined according to the method of Broderica and Kang (21). MCP concentration was analyzed according to the method of Makkar et al. (22).

### Determination of Apparent Nutrient Digestibility

Feces were collected for five consecutive days at the end of the trial. In the laboratory, all feces of each replicate were evenly mixed and 10% sulfuric acid was added to fix nitrogen. Fecal samples were stored at −20^°^C for subsequent determination.

Dry matter (DM), crude protein (CP), ash, and ether extract (EE) of the feed samples and fecal samples were determined according to the Association of Analytical Communities (AOAC) (23) using the methods 967.03, 984.13, 924.05, and 920.39, respectively. Neutral detergent fiber (NDF) and acid detergent fiber (ADF) were determined using an Ankom A200i Fiber analyzer (ANKOM Technology Co., New York, NY, United States) according to Van Soest et al. (24). The content of hydrochloric acid insoluble ash (AIA) was determined according to Keulen and Young (25) described, and AIA was used as a digestibility marker. The formula was:


AD(%)=[(a/c-b/d)/(a/c)]×100,

where *AD* represents the apparent digestibility of the nutrient content, *a* represents the nutrient content of feedstuffs, *b* represents the nutrient content of feces, *c* represents the AIA content of feedstuffs, and *d* represents the AIA content of feces.

### Determination of Slaughter Performance

One sheep was randomly selected from each replicate for slaughter, after fasting for 12 h. After the removal of head, feet, tail, and internal organs, the carcass, heart, spleen, liver, lungs, and kidneys were weighed. The dressing percentage was calculated as: carcass weight (kg)/live weight (kg) before slaughter × 100%. The organ index was calculated as organ index = organ weight (g)/live weight (kg) before slaughter. Backfat thickness, eye muscle area (EMA), and grade-rule (GR) tissue depth values were measured on the left side of the carcass according to Song et al. (26). Backfat thickness is the thickness between the 12th and 13th ribs. EMA is the cross-sectional area of the 12th and 13th ribs. GR is the total tissue thickness between the 12th and 13th rib and 11 cm from the dorsal midline of the carcass, which can represent the carcass fat content.

### Statistical Analysis

All data were analyzed using SPSS 17.0 software (SPSS Inc., Chicago, IL, United States). Data were analyzed using double-tailed *t*-tests after checking normal distribution. The data were considered significantly different if *p* ≤ 0.05, and a tendency was suggested if 0.05 < *p* < 0.10.

## Results

### Temperature and Humidity Index Values

**FIGURE 1:**
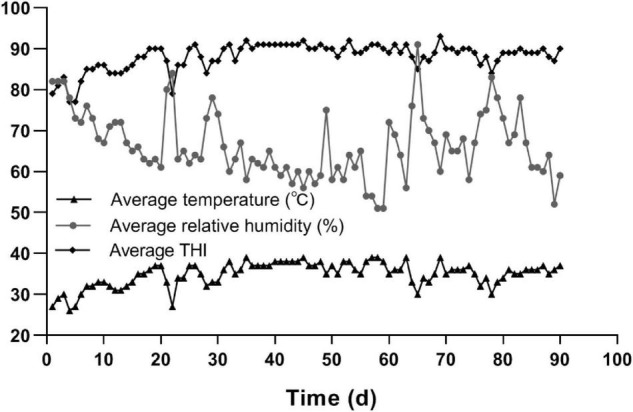
The average temperature, average relative humidity, and *THI* during the trial period.

The Hu sheep was suffering from heat stress throughout the whole trial period, as shown in Figure 1. The days that the Hu sheep spent at different *THI* thresholds were: 72 ≤ *THI <* 78 for 2 days, 78 ≤ *THI* < 90 for 48 days, and *THI*s ≥ 90 for 40 days.

### Growth Performance

The growth performance of the Hu sheep is shown in Table 2. No differences were observed in body weight on days 30, 60, and 90 between CON and GLU (*p* > 0.05). No differences were observed in ADFI, ADG, and F:G ratio between GLU and CON during phase 1 (1–30 days) and phase 2 (31–60 days) (*p* > 0.05). However, during phase 3 (61–90 days), the ADG in GLU was higher than in CON (*p <* 0.05), and the ADFI and F:G ratio in GLU was lower, compared with those in CON (*p <* 0.05).

**TABLE 2:** Effects of glutamate supplementation on growth performance of heat-stressed Hu sheep (*n* = 6)^1^.

Items	CON	GLU	SEM	*P-*value
**Body weight (BW)**				
Day 0, kg	17.77	17.71	0.173	0.877
Day 30, kg	24.70	24.78	0.353	0.912
Day 60, kg	31.08	31.31	0.449	0.800
Day 90, kg	36.13	37.02	0.501	0.379
**Phase 1, 1–30** **days**				
ADFI, g/d	1110.53	1108.67	13.140	0.944
ADG, g/d	231.11	235.56	8.491	0.797
F:G	4.81	4.71	0.220	0.810
**Phase 2, 31–60 days**				
ADFI, g/d	1343.00	1358.00	7.903	0.347
ADG, g/d	212.78	217.85	6.798	0.714
F:G	6.31	6.23	0.216	0.870
**Phase 3, 61–90 days**				
ADFI, g/d	1465.67^a^	1436.00^b^	6.976	0.032
ADG, g/d	168.26^b^	190.35^a^	5.484	0.043
F:G	8.71^a^	7.54^b^	0.259	0.021
**Whole phase, 1–90 days**				
ADFI, g/d	1306.40	1300.89	12.504	0.820
ADG, g/d	204.05	214.58	4.577	0.254
F:G	6.40	6.06	0.143	0.246

*^1^CON, basal diet group; GLU, basal diet + 3 g/head/day glutamate group.*

*ADFI, average daily feed intake; ADG, average daily gain; F:G, ADFI/ADG.*

*^a,b^Different superscripts indicate significant differences within a row (P < 0.05).*

### Slaughter Performance

Table 3 shows no differences in carcass weight, dressing percentage, backfat thickness, eye muscle area, GR value, organ weight, and organ index between GLU and CON (*p >* 0.05). However, spleen weight (*p* = 0.078) and spleen index (*p* = 0.079) in GLU tended to increase (by 54.11 and 53.71%), respectively, as compared with CON (Table 4).

**TABLE 3:** Effects of glutamate supplementation on carcass traits of heat stressed Hu sheep (*n* = 6)^1^.

Items	CON	GLU	SEM	*P-*value
Live weight before slaughter, kg	37.11	37.05	0.343	0.915
Carcass weight, kg	18.57	18.53	0.257	0.940
Dressing percentage^2^, %	50.06	49.97	0.598	0.946
**Carcass traits**				
Backfat thickness^3^, mm	15.98	16.15	0.228	0.258
Eye muscle area^4^, cm^2^	24.70	24.78	0.899	0.929
GR value^5^, mm	18.61	19.00	0.352	0.609

*^1^CON, basal diet group; GLU, basal diet + 3 g/head/day glutamate group.*

*^2^Dressing percentage = carcass weight/live weight before slaughter.*

*^3^Between the 12th and 13th ribs on the left side of the carcass.*

*^4^The cross-sectional areas of the 12th and 13th rib were depicted with sulfate paper. Then the area was calculated by the formula: eye muscle area = height × width × 0.7.*

*^5^Grade-rule (GR) value = the total tissue thickness between the 12th and 13th ribs and 11 cm from the dorsal midline of the carcass.*

*^a,b^Different superscripts indicate significant differences within a row (P < 0.05).*

**TABLE 4:** Effects of glutamate supplementation on organ index of heat-stressed Hu sheep (*n* = 6)^a^.

Items	CON	GLU	SEM	*P-*value
**Organ weight**				
Heart, g	127.41	127.09	2.546	0.954
Spleen, g	51.62	79.55	7.498	0.078
Liver, g	611.21	588.73	14.984	0.479
Kidney, g	98.64	99.09	2.222	0.925
Lung, g	341.57	376.69	13.224	0.197
**Organ index^b^**				
Heart, g/kg	3.44	3.43	0.073	0.966
Spleen, g/kg	1.40	2.15	0.203	0.079
Liver, g/kg	16.46	15.91	0.396	0.510
Kidney, g/kg	2.66	2.68	0.066	0.916
Lung, g/kg	9.22	10.16	0.356	0.196

*^a^CON, basal diet group; GLU, basal diet + 3 g/head/day glutamate group.*

*^b^Organ index = organ weight (g)/live weight before slaughter (kg).*

### Apparent Nutrient Digestibility

As shown in Table 5, the digestibility of DM, OM, and CP in GLU was higher than those in CON (*p <* 0.05), whereas no significant differences were observed in the digestibility of EE, NDF, ADF, and soluble carbohydrates (SCHO) between GLU and CON (*p* > 0.05).

**TABLE 5:** Effects of glutamate supplementation on apparent nutrient digestibility of heat-stressed Hu sheep (*n* = 6)^1^.

Items	CON	GLU	SEM	*P-*value
DM, %	82.30^b^	83.62^a^	0.318	0.030
OM, %	85.27^b^	86.58^a^	0.290	0.015
SCHO, %	86.82	89.52	1.382	0.352
CP, %	71.46^b^	74.61^a^	0.624	0.004
EE, %	77.20	77.25	0.289	0.938
NDF, %	64.44	65.35	0.625	0.497
ADF, %	57.70	60.23	0.828	0.131

*^1^CON, basal diet group; GLU, basal diet + 3 g/head/day glutamate group; M, dry matter; OM, organic matter; CP, crude protein; EE, ether extract; NDF, neutral detergent fiber; ADF, acid detergent fiber; SCHO, soluble carbohydrates.*

*^a,b^Different superscripts indicate significant differences within a row (P < 0.05).*

### Rumen Fermentation Characteristics

Rumen fermentation characteristics are shown in Table 6. Ruminal pH, MCP, NH_3_-N, and isovalerate concentration in GLU were all higher than those in CON (*p <* 0.05). The concentrations of TVFAs and VFAs in GLU, except for isovalerate, were similar to those in CON (*p >* 0.05).

**TABLE 6:** Effects of glutamate supplementation on rumen fermentation characteristics of heat-stressed Hu sheep (*n* = 6)^1^.

Items	CON	GLU	SEM	*P-*value
pH	6.11^b^	6.44^a^	0.086	0.050
MCP^2^, mg/dL	261.10^b^	381.71^a^	27.103	0.012
NH_3_-N^3^, mg/dL	15.23*^b^*	19.91^a^	1.111	0.027
Acetate, mmol/L	36.25	37.02	3.525	0.920
Propionate, mmol/L	7.41	8.67	0.677	0.383
Butyrate, mmol/L	8.87	7.90	1.007	0.655
Isobutyrate, mmol/L	0.55	0.92	0.110	0.093
Valerate, mmol/L	0.63	0.77	0.063	0.310
Isovalerate, mmol/L	1.05^b^	1.76^a^	0.154	0.011
Acetate/Propionate, A/P	4.82	4.25	0.161	0.075
TVFA^4^, mmol/L	54.76	57.04	5.316	0.844

*^1^CON, basal diet group; GLU, basal diet + 3 g/head/day glutamate group.*

*^2–4^MCP, microbial crude protein; NH_3_-N, ammonia nitrogen; TVFA, total volatile fatty acids, calculated as follows: acetate + propionate + butyrate + isobutyrate + valerate + isovalerate.*

*^a,b^Different superscripts indicate significant differences within a row (P < 0.05).*

### Serum Biochemical Indices and Serum Hormones

The levels of serum glutamate, GLB, IgA, IgG, and IgM in GLU were higher than in CON on day 90 (*p <* 0.05), and the ALB/GLB in GLU was lower than that in CON on day 90 (*p <* 0.05) (Table 7). However, all the serum biochemical parameters-listed above were similar between GLU and CON on days 30 and 60 (*p >* 0.05).

**TABLE 7:** Effects of glutamate supplementation on serum biochemistry of heat-stressed Hu sheep (*n* = 6)^1^.

Items		CON	GLU	SEM	*P-*value
Glutamate, μmol/L	30*day*	148.89	149.32	2.334	0.931
	60*day*	143.57	143.68	2.422	0.983
	90*day*	144.90^b^	155.29^a^	2.433	0.024
Glucose, mmol/L	30*day*	2.76	3.14	0.166	0.290
	60*day*	3.30	3.53	0.160	0.487
	90*day*	1.44	1.54	0.199	0.832
BUN, mmol/L	30*day*	4.97	5.41	0.162	0.185
	60*day*	6.03	6.26	0.276	0.706
	90*day*	7.21	7.23	0.167	0.942
TP, g/L	30*day*	71.84	74.04	1.443	0.471
	60*day*	74.70	75.09	0.883	0.834
	90*day*	74.46	77.28	0.827	0.088
ALB, g/L	30*day*	32.86	32.44	0.542	0.375
	60*day*	39.17	37.80	0.595	0.267
	90*day*	39.57	39.10	0.468	0.641
GLB, g/L	30*day*	38.98	41.61	1.596	0.430
	60*day*	35.52	37.30	0.908	0.353
	90*day*	34.90^b^	38.18^a^	0.719	0.013
ALB/GLB	30*day*	0.85	0.80	0.037	0.555
	60*day*	1.11	1.03	0.038	0.291
	90*day*	1.14^a^	1.03^b^	0.026	0.025
IgA, g/L	30*day*	1.63	1.64	0.019	0.715
	60*day*	1.56	1.50	0.031	0.316
	90*day*	1.54^b^	1.70^a^	0.030	0.008
IgG, g/L	30*day*	16.76	16.53	0.317	0.731
	60*day*	16.22	16.15	0.327	0.926
	90*day*	16.14^b^	17.69^a^	0.374	0.030
IgM, g/L	30*day*	1.31	1.19	0.063	0.365
	60*day*	1.20	1.11	0.046	0.339
	90*day*	1.18^b^	1.34^a^	0.041	0.037

*^1^CON, basal diet group; GLU, basal diet + 3 g/head/day glutamate group.*

*BUN, blood urea nitrogen; TP, total protein; ALB, albumin; GLB, globulin; IgA, immunoglobulin A; IgG, immunoglobulin G; IgM, immunoglobulin M.*

*^a,b^Different superscripts indicate significant differences within a row (P < 0.05).*

The levels of serum hormones and HSP70 are shown in Table 8. The level of GH in GLU was higher than those in CON on day 90 (*p* = 0.001), and the levels of HSP70, ACTH, CORT, T_3_, and T_4_ in GLU were lower than those in CON on day 90 (*p <* 0.001). However, no differences were found between GLU and CON on days 30 and 60 (*p >* 0.05), expect that level of T_3_ was lower in GLU on day 30 (*p <* 0.05).

**TABLE 8:** Effects of glutamate supplementation on serum hormone and HSP70 of heat-stressed Hu sheep (*n* = 6)^1^.

Items		CON	GLU	SEM	*P-*value
HSP70, pg/mL	30*d*	210.57	214.13	2.412	0.502
	60*d*	220.23	232.52	3.570	0.100
	90*d*	240.37^a^	184.36^b^	8.855	<0.001
GH, ng/mL	30*d*	4.83	5.40	0.217	0.207
	60*d*	4.65	4.76	0.087	0.560
	90*d*	4.28^b^	5.32^a^	0.185	0.001
ACTH, pg/mL	30*d*	16.11	19.51	1.071	0.116
	60*d*	19.88	19.82	0.579	0.967
	90*d*	22.01^a^	15.51^b^	1.018	<0.001
CORT, ng/mL	30*d*	46.04	53.32	2.629	0.203
	60*d*	51.46	51.49	3.153	0.996
	90*d*	62.68^a^	43.25^b^	3.150	<0.001
T_3_, ng/mL	30*d*	1.19^a^	1.09^b^	0.024	0.026
	60*d*	1.14	1.20	0.025	0.199
	90*d*	1.24^a^	1.05^b^	0.030	<0.001
T_4_, ng/mL	30*d*	62.43	67.00	1.584	0.159
	60*d*	65.55	70.80	3.258	0.460
	90*d*	91.72^a^	62.79^b^	5.106	<0.001

*^1^CON, basal diet group; GLU, basal diet + 3 g/head/day glutamate group.*

*GH, growth hormone; HSP70, heat shock protein 70; ACTH, adrenocorticotrophic hormone; CORT, corticosterone; T_3_, triiodothyronine; T_4_, thyroxine.*

*^a,b^Different superscripts indicate significant differences within a row (P < 0.05).*

## Discussion

Temperature and humidity index is widely used as a practical indicator of the degree of heat stress. Thornton et al. (27) summarized that the *THI* threshold for heat stress in sheep was as follows: absence of heat stress (*THI <* 72), moderate heat stress (72 ≤ *THI <* 78), high heat stress (78 *≤ THI <* 90), and extreme heat stress (*THI* ≥ 90). Li et al. (28) indicated that Hu sheep suffered from heat stress when they were exposed to *THI*_*M*_ ≥ 75 conditions. In the present study, the Hu sheep spent the whole period of 90 days in *THI* ≥ 75 conditions. It suggested that the Hu sheep suffered from either high or extreme heat stress throughout the whole trial period, especially during phases 2 and 3.

### Glutamate Improves Growth Performance of Heat-Stressed Hu Sheep

It is known that the heat stress leads to decreases in feed intake, nutrient digestibility, and absorption capacity, resulting in reduced animal growth performance of livestock (29). The research results of glutamate on the growth performance of animals are inconsistent. Wang et al. (30) found that the glutamate was a key neurotransmitter in the hypothalamus, which could inhibit the feed intake in animals. Other researchers indicated that it could also increase the feed intake by activating taste receptors in the digestive system (31). Hu et al. (32) and Rezaei et al. (33) reported lower ADFI after glutamate supplementation in pigs. In growing-finishing pigs, glutamate supplementation of 1% had no effect on ADG and F:G for the 60 days feeding regimen (32). By contrast, it linearly increased ADG, and decreased ADFI and F:G with monosodium glutamate addition of 0.5–4% in post-weaning pigs (33). In dairy calves, a lower dosage of glutamate (0.1 or 0.3%) has no obvious effect on ADFI, ADG, and F:G within a 70 or 56 days feeding period (34, 35). In this study, similar results of glutamate supplementation were shown in Hu sheep during phase 1 (1–30 days) and phase 2 (31–61 days). However, glutamate resulted in a marked increase of ADG, and a decrease of ADFI and F:G during phase 3 (61–90 days), which might be because of the long experimental period of glutamate supplementation. The levels of serum glutamate, serum biochemical indices and serum hormones were similar during phases 1 and 2, whereas a higher level of serum glutamate, GLB, IgA, IgG, IgM, and GH, and a lower level of HSP70, ACTH, CORT, T_3_, and T_4_ in GLU were observed during phase 3 in this study. It suggested that a longer period of glutamate supplementation increased the serum glutamate to promote the growth performance, improve the metabolism, and enhance the immunity of Hu sheep.

### Glutamate Improves Rumen Fermentation

Accounting for the highest proportion of AA composition in rumen microorganisms, glutamate plays an essential role in MCP synthesis and ruminant growth (36). In this study, a higher concentration of MCP was observed owing to the dietary supplementation of glutamate, which promoted rumen microbial growth (16, 17). The NH_3_-N is the main source of nitrogen for rumen microbial protein synthesis, and its utilization rate reflects the balance between substrate nitrogen degradation and microbial synthesis utilization (37). In the present study, the NH_3_-N concentration was notably increased by adding glutamate, which was consistent with a previous report demonstrating that dietary glutamate addition increased the NH_3_-N concentration in dairy cows (38). In this study, higher levels of NH_3_-N *via* the supplementation with glutamate provided a nitrogen source promoting microbial growth and reproduction, which supported the higher MCP in GLU. The higher NH_3_-N concentration in GLU indicated a higher rumen fermentation rate; however, the NH_3_-N concentration in this study did not reach the 235 mg/L required for the maximal fermentation rate (39), suggesting that the specific dosage of glutamate addition did not inhibit the rumen fermentation in Hu sheep. The concentrations of NH_3_-N and VFAs are considered to be the main factors affecting ruminal pH. In the present study, dietary glutamate supplementation increased rumen pH, which might be attributed to the higher NH_3_-N production in GLU. The results were consistent with the study of Dann et al. (38) who reported that glutamate supplementation could increase the rumen pH. VFAs are absorbed by the ruminal epithelial cells and provide the main energy substances for the ruminant production. These organic acids can improve the morphology of rumen mucosa, and reduce the infection of intestinal diseases by improving the intestinal microbial structure and morphology (40, 41, 42). Isovalerate is also an energy substance for the ruminal epithelial cells, and linear increases in isovalerate are positively correlated with the promotion of rumen development (40). In this study, the higher isovalerate in GLU might be associated with an improved feed degradation rate.

### Glutamate Improves Nutrient Digestibility of Heat-Stressed Hu Sheep

Oral intake of glutamate stimulates the salivation essential for mastication, swallowing, and digestion (43). A previous study demonstrated that free glutamate in digestive juices obtained from dietary digestion, absorption, or glutamine synthesis, could improve nutrient digestibility by stimulating the release of digestive enzymes and pancreatic hormones (44). Glutamate also could improve intestinal morphology and promote nutrient utilization in heat-stressed broilers (13). In the present study, the higher digestibility of DM, OM, and CP in GLU was probably due to higher serum glutamate, as mentioned above. The results of this study were similar to the research of Dann et al. (38) who reported that supplementation of 80 g glutamate per cow/day tended to increase the digestibility of DM and OM.

### Glutamate Improves Immunity of Heat-Stressed Hu Sheep

Heat stress reduces animal immune function, such as decreased immune antibody secretions and spleen developmental retardation (45, 46). As the largest secondary lymphoid organ, the spleen is rich in various immune cells, such as B cells and T cells, which play vital roles in immune function and are responsible for IgA, IgG, and IgM production (47, 48). In the present study, glutamate supplementation tended to increase the spleen weight and the spleen index by 54.11 and 53.71%, respectively, probably because glutamate was beneficial to spleen development through the activation of T cell function and protection against T cell apoptosis (49). It is critical to emphasize that glutamate is an important immunomodulator (50). A previous study reported that the glutamate receptors (GluRs) were highly expressed in T cells and B cells (51), suggesting that glutamate had a positive effect on targeting them. However, if serum glutamate concentration exceeds the pathological 10^–3^ moles level, then T cell function and survival are inhibited (49). Serum glutamate in this study was within normal physiological concentrations. As mentioned above, higher levels of IgA, IgG, and IgM in GLU might result from the higher serum glutamate in GLU stimulating spleen development and activating T cell function. Furthermore, the decreased level of ALB/GLB in GLU might be because of the increased GLB concentration.

### Glutamate Improves Serum Hormone and HSP70 Levels in Heat-Stressed Hu Sheep

The hypothalamic–pituitary–adrenal (HPA) axis and hypothalamic–pituitary–thyroid (HPT) axis can be activated by heat stress, which in turn regulate the corresponding hormones secreted by endocrine glands (52). Higher secretion of serum ACTH and CORT has been reported in animals exposed to heat stress (53). Generally, CORT is an appropriate end-product of the HPA axis, and higher ACTH leads to increase CORT (54). However, excessive CORT would accelerate the heat stress-induced protein degradation (55), which partially explained the lower body weight in CON in this experiment. The lower levels of ACTH and CORT in GLU on day 90 might result from the higher level of serum glutamate, which reduced the secretion of ACTH and CORT and the protein degradation of muscle.

The T_3_ and T_4_ (the main thyroid hormones), are key regulators of energy metabolism in the body (56). It is essential for animals to maintain the thermal balance by lowering the secretion of T_3_ and T_4_ (56). Several studies have reported that monosodium glutamate has a cumulative effect on reducing thyroid function (57, 58). Similarly, in this study, a decrease in T_3_ and T_4_ after dietary glutamate supplementation was observed on day 90, suggesting that dietary glutamate supplementation might reduce heat stress responses by lowering heat production. A higher GH level often indicates a better anti-heat stress capability in goats (59). As an excitatory AA, glutamate contributes to the stimulation of GH secretion by regulating the hypothalamus (60). Serum GH concentrations are linearly correlated with serum glutamate (60), which can explain the higher level of GH in GLU in this study.

The HSP70, the most widely distributed and expressed HSP in the body, is involved in regulating the stability of cellular proteins, increasing the tolerance of cells to stressors, and reducing cell apoptosis (61). Higher HSP70 is usually positively related to the degree of heat stress (62). A previous study reported that glutamate was conducive to enhancing the thermodynamic stability of proteins against temperature stress (12). Another study showed that glutamine normalized metabolism by reducing the serum levels of HSP70 in atrial fibrillation patients (63). In the present study, HSP70 content in GLU decreased on day 90, suggesting that glutamate supplementation might reduce the heat stress response and normalize metabolism by reducing HSP70 levels.

## Conclusion

In the present study, we found that a longer period of dietary glutamate supplementation (not less than 60 days) at a dosage of 3 g/head/day was beneficial to heat-stressed Hu sheep. It might improve the growth performance of the animals by increasing the level of serum glutamate to promote growth hormone level, decrease heat stress-related hormones and HSP70 levels, and enhance immune function. These findings suggest that a strategy of glutamate supplementation has important advantages in the sheep industry under heat stress in summer.

## Data Availability Statement

The original contributions presented in the study are included in the article/supplementary material, further inquiries can be directed to the corresponding authors.

## Ethics Statement

The animal care and experimental procedures in this study were approved by the Animal Ethics Committee of the Jiangxi Agricultural University (JXAULL-2021-036).

## Author Contributions

CL, KO, and QQ designed the experiments. CL and JZ carried out the experiments and performed the analysis. CL drafted the manuscript. YL, XZ, HL, KO, and QQ revised the manuscript. KL provided the feeding grounds for the experiment. All authors contributed to the article preparation and approved the submitted version.

## Conflict of Interest

KL was employed by Ganzhou Lvlinwan Agriculture and Animal Husbandry Co. Ltd. The remaining authors declare that the research was conducted in the absence of any commercial or financial relationships that could be construed as a potential conflict of interest.

## Publisher’s Note

All claims expressed in this article are solely those of the authors and do not necessarily represent those of their affiliated organizations, or those of the publisher, the editors and the reviewers. Any product that may be evaluated in this article, or claim that may be made by its manufacturer, is not guaranteed or endorsed by the publisher.
